# OsWRKY114 Is a Player in Rice Immunity against *Fusarium fujikuroi*

**DOI:** 10.3390/ijms24076604

**Published:** 2023-04-01

**Authors:** Giha Song, Seungmin Son, Suhyeon Nam, Eun-Jung Suh, Soo In Lee, Sang Ryeol Park

**Affiliations:** 1 National Institute of Agricultural Sciences, Rural Development Administration, Jeonju 54874, Republic of Korea; 2 Department of Crop Science & Biotechnology, Jeonbuk National University, Jeonju 54896, Republic of Korea

**Keywords:** *Fusarium fujikuroi*, gibberellin, innate immunity, jasmonic acid, OsWRKY114, rice

## Abstract

Every year, invasive pathogens cause significant damage to crops. Thus, identifying genes conferring broad-spectrum resistance to invading pathogens is critical for plant breeding. We previously demonstrated that OsWRKY114 contributes to rice (*Oryza sativa* L.) immunity against the bacterial pathovar *Xanthomonas oryzae* pv. *oryzae* (*Xoo*). However, it is not known whether OsWRKY114 is involved in defense responses to other pathogens. In this study, we revealed that OsWRKY114 enhances innate immunity in rice against the fungal pathogen *Fusarium fujikuroi*, which is the causal agent of bakanae disease. Transcript levels of various gibberellin-related genes that are required for plant susceptibility to *F. fujikuroi* were reduced in rice plants overexpressing *OsWRKY114*. Analysis of disease symptoms revealed increased innate immunity against *F. fujikuroi* in *OsWRKY114*-overexpressing rice plants. Moreover, the expression levels of *OsJAZ* genes, which encode negative regulators of jasmonic acid signaling that confer immunity against *F. fujikuroi*, were reduced in *OsWRKY114*-overexpressing rice plants. These results indicate that OsWRKY114 confers broad-spectrum resistance not only to *Xoo* but also to *F. fujikuroi*. Our findings provide a basis for developing strategies to mitigate pathogen attack and improve crop resilience to biotic stress.

## 1. Introduction

Global crop production is severely impaired by invading pathogens, including bacteria and fungi. The recent rapid changes in climate have exacerbated the damage caused by plant diseases [1]. Therefore, to support worldwide food demands, an important agricultural goal is to conserve crop yields threatened by pathogen attack. Rice (*Oryza sativa* L.) is a major staple crop, providing a source of nutrients for over half the world’s population [2]. Thus, identifying novel genes conferring broad-spectrum resistance (BSR) in rice can play a significant role in ensuring crop and nutritional security.

The necrotrophic fungus *Fusarium fujikuroi* is the causal agent of bakanae disease, leading to losses of up to 95% in rice yields [3]. This damage is predicted to be exacerbated by climate change [4]. Therefore, identifying and investigating defense genes involved in the rice–*F. fujikuroi* interaction are important for sustaining yields and improving crop resilience. Phytohormones such as gibberellin (GA) and jasmonic acid (JA) play central roles in the rice–*F. fujikuroi* relationship. GA is synthesized by all vascular plants as well as some bacteria and fungi, including *F. fujikuroi* [5]. GA controls processes involved in plant growth and development, including seed germination; GA promotes seed germination, while abscisic acid (ABA) inhibits this process [6]. Additionally, many studies have shown that GA is involved in abiotic and biotic stress responses [7,8]. Direct interactions between DELLA proteins (which are negative regulators of GA signaling) and Jasmonate ZIM domain (JAZ) proteins (JA signaling repressors) result in antagonistic GA-JA crosstalk that helps coordinate plant growth and defense [9]. JAZ proteins play central roles in the JA signaling cascade, which is activated upon decreases in JAZ protein levels [10]. JA signaling is important for various plant processes [11,12]. In particular, JA-dependent responses are major contributors to innate immunity against necrotrophic pathogens, including *F. fujikuroi* [13]. High-performance liquid chromatography-mass spectrometry and comparative transcriptome profiling showed that increasing GA and decreasing JA levels in infected plants are important for plant susceptibility to *F. fujikuroi* [14,15]. Transcriptome analysis revealed that the expression levels of *JAZ* genes decrease during the early stages of *F. fujikuroi* infection and that JA-dependent immunity is crucial for the resistance of rice to *F. fujikuroi* [16]. However, the signaling components and regulatory mechanisms of GA and JA signaling involved in rice–*F. fujikuroi* interactions are largely unknown.

WRKY transcription factors play key roles in various plant defense responses [17]. WRKY proteins contain one or two WRKY domains consisting of a highly conserved WRKY motif (WRKYGQK) and a zinc finger DNA-binding region [18]. WRKY Group III transcription factors, which contain one WRKY domain with a C2HC zinc finger region, are known as the most evolved WRKYs and are primarily involved in innate immunity [19,20,21,22]. We previously demonstrated that the rice WRKY Group III transcription factor OsWRKY114, a transcriptional activator, increases resistance to *Xanthomonas oryzae* pv. *oryzae* (*Xoo*) by inducing pathogen-related (*PR*) gene expressing and suppressing ABA signaling [23,24]. However, the roles of OsWRKY114 in defense responses to other pathogens are unknown. Here, we demonstrate that OsWRKY114 increases resistance to *F. fujikuroi* by modulating GA and JA signaling.

## 2. Results

### 2.1. OsWRKY114 Represses GA Signaling in Rice

**Figure 1:**
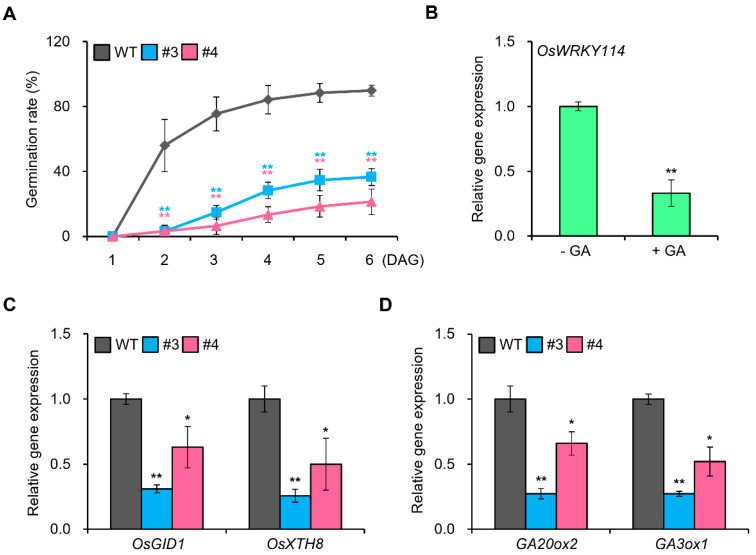
OsWRKY114 is involved in GA responses. (**A**) Germination rate of *OsWRKY114_OX_*. Germination rate was measured in 36 kernels each from two *OsWRKY114_OX_* plants (#3 and #4) and one wild-type (WT) plant grown on half-strength MS medium in a growth room under continuous light at 28 °C for 6 days. Values are expressed as means ± SD. Asterisks indicate values significantly different from those of the WT (** *p* < 0.01). DAG, days after germination. (**B**) Relative expression levels of *OsWRKY114* in WT plants after GA treatment. WT plants were grown on MS medium with or without 1 μM GA_3_ for 6 DAG. The transcript levels of *OsWRKY114* were analyzed by RT-qPCR. *OsActin* was used as an internal control to quantify relative gene expression. Values are expressed as means ± SD. Asterisks indicate values significantly different from those of the non-GA-treated control (** *p* < 0.01). (**C**,**D**) Relative expression levels of GA-related genes in *OsWRKY114_OX_*. Total RNA was extracted from 6-day-old *OsWRKY114_OX_* and WT seedings. Transcript levels of genes involved in GA signaling (**C**) and GA biosynthesis (**D**) were analyzed using RT-qPCR. *OsActin* was used as an internal control to quantify relative gene expression. Values are expressed as means ± SD. Asterisks indicate values significantly different from those of the WT control (* *p* < 0.05 and ** *p* < 0.01). All experiments were repeated at least three times with similar results. Representative graphs are shown.

Since OsWRKY114 negatively regulates ABA signaling [24,25], we expected that overexpressing *OsWRKY114* would promote seed germination. To investigate this notion, we measured germination rates in transgenic rice plants constitutively expressing *OsWRKY114* (*OsWRKY114_OX_*) and in wild-type plants. Surprisingly, the rate of seed germination was slower in *OsWRKY114_OX_* than in wild-type plants (Figure 1A). Based on this observation, we hypothesized that OsWRKY114 also represses GA signaling, which is required for germination. To determine whether OsWRKY114 is involved in GA signaling, we monitored the expression levels of *OsWRKY114* in wild-type plants after GA treatment. *OsWRKY114* transcript levels decreased in response to GA treatment (Figure 1B). To further test our hypothesis, we performed reverse-transcription quantitative PCR (RT-qPCR) to analyze the transcript levels of various genes involved in GA signaling or biosynthesis in *OsWRKY114_OX_* plants. The expression levels of *GIBBERELLIN-INSENSITIVE DWARF 1* (*OsGID1*) and *XYLOGLUCAN ENDOTRANSGLYCOSYLASE/HYDROLASE 8* (*OsXTH8*) were significantly reduced in *OsWRKY114_OX_* compared with wild-type plants (Figure 1C). OsGID1 is a GA receptor that triggers GA signaling [26], and OsXTH8 is a specific marker of GA responses [27,28]. Therefore, the downregulation of *OsGID1* and *OsXTH8* in *OsWRKY114_OX_* strongly suggests that OsWRKY114 suppresses GA signaling in rice. Moreover, gene expression analysis showed that the transcript levels of *GA 20-OXIDASE 2* (*GA20_OX_2*) and *GA3_OX_1*, encoding two key enzymes that regulate GA biosynthesis, were reduced in *OsWRKY114_OX_* compared with wild-type plants (Figure 1D). Taken together, these results indicate that OsWRKY114 negatively regulates GA signaling.

### 2.2. Resistance to F. fujikuroi Is Enhanced in OsWRKY114-Overexpressing Plants

**Figure 2:**
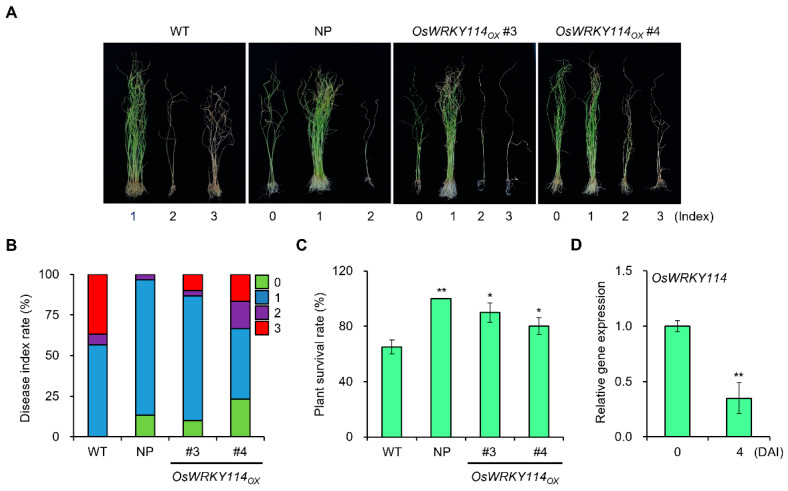
OsWRKY114 increases resistance to *Fusarium fujikuroi*. (**A**–**C**) Bakanae disease assay of *OsWRKY114_OX_*. Four-day-old *OsWRKY114_OX_*, bakanae disease-resistant variety Nampyeong (NP), and wild-type (WT) seedlings were inoculated with *F. fujikuroi* CF283. Images were acquired 21 days after inoculation (**A**) and disease index (**B**) and survival rate (**C**) were calculated. Disease index: 0, no symptoms appeared; 1, blight symptoms appeared on leaf tips; 2, shoots wilted and leaf color changed to pale green; 3, the whole leaf dried out. Values represent means ± SD. Asterisks indicate values significantly different from those of the WT control (* *p* < 0.05 and ** *p* < 0.01). (**D**) Relative expression levels of *OsWRKY114* after *F. fujikuroi* inoculation. Four-day-old WT seedlings were inoculated with *F. fujikuroi* CF283 for 4 days. Total RNA was extracted from the seedlings before and after *F. fujikuroi* inoculation. Transcript levels of *OsWRKY114* were analyzed by RT-qPCR. *OsActin* was used as an internal control to quantify relative gene expression. Values are expressed as means ± SD. Asterisks indicate values significantly different from those of the mock-inoculated control (** *p* < 0.01). All experiments were repeated at least three times, and similar results were obtained. Representative graphs are shown.

GA accumulation and responses in rice are key contributors to susceptibility to *F. fujikuroi* [14,15]. Therefore, we inoculated 4-day-old *OsWRKY114_OX_* and wild-type seedlings with a compatible strain of *F. fujikuroi* (CF283) and analyzed disease symptoms 21 days after inoculation (DAI). We used the well-known bakanae disease-resistant rice variety Nampyeong as a positive control [29]. In addition, we performed detailed analysis using the disease index method [30]. Although *OsWRKY114_OX_* lines showed weaker resistance to *F. fujikuroi* compared with Nampyeong, the bakanae disease index score indicated that overexpression of *OsWRKY114* in the susceptible cultivar increases the resistance compared with the wild-type plant (Figure 2A,B). Moreover, the survival rates of *OsWRKY114_OX_* plants after *F. fujikuroi* inoculation were much higher than those of wild-type plants (Figure 2C). These results demonstrate that overexpressing *OsWRKY114* enhances innate immunity against *F. fujikuroi*. To examine whether *OsWRKY114* expression is a natural response to *F. fujikuroi* invasion, we inoculated wild-type plants with *F. fujikuroi* and analyzed *OsWRKY114* transcript levels at early stages of infection (4 DAI). Wild-type plants inoculated with *F. fujikuroi* showed reduced *OsWRKY114* transcript levels (Figure 2D), suggesting that inhibited *OsWRKY114* expression is important for susceptibility of *F. fujikuroi*.

### 2.3. OsWRKY114 Increases JA Responses by Inhibiting JAZ Gene Expression

**Figure 3:**
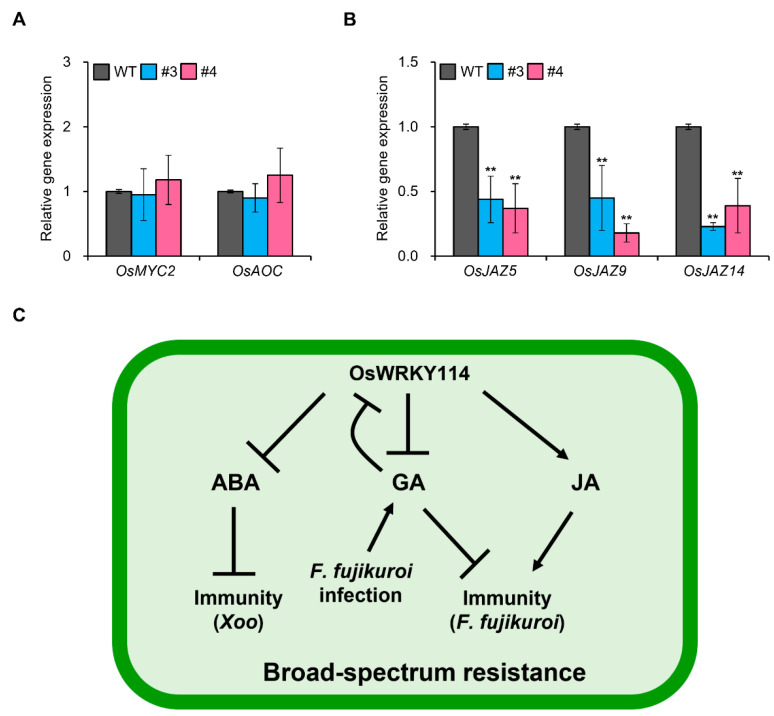
OsWRKY114 decreases *OsJAZ* gene expression. (**A**,**B**) Relative expression levels of JA-related genes in *OsWRKY114_OX_*. Total RNA was extracted from 6-day-old *OsWRKY114_OX_* and WT seedlings. The transcript levels of genes encoding positive regulators (**A**) and negative regulators (**B**) of JA signaling were analyzed via RT-qPCR. *OsActin* was used as an internal control to quantify relative gene expression. Values are expressed as means ± SD. Asterisks indicate values significantly different from those of the WT control (** *p* < 0.01). All experiments were repeated at least three times, with similar results. Representative graphs are shown. (**C**) A working model of OsWRKY114-induced broad-spectrum resistance via the regulation of phytohormone signaling. OsWRKY114-mediated inhibition of ABA signaling increases resistance to *Xanthomonas oryzae* pv. *oryzae*, while downregulating GA and upregulating JA signaling via OsWRKY114 enhances resistance to *Fusarium fujikuroi*.

JA-dependent defense responses are important factors in resistance to *F. fujikuroi* in rice [14,16]. To determine whether OsWRKY114 is also involved in JA responses, we analyzed the expression levels of various JA-related genes in *OsWRKY114_OX_*. *MYC2* and *AOC*, which encode positive regulators of JA signaling and JA biosynthesis [31,32], did not show significant differences in expression in *OsWRKY114_OX_* vs. wild-type plants (Figure 3A). We then measured the expression levels of genes encoding negative regulators of JA signaling, including *JAZ* genes. Interestingly, the transcript levels of *OsJAZ5*, *OsJAZ9*, and *OsJAZ14* were significantly reduced in *OsWRKY114_OX_* compared with wild-type plants (Figure 3B). These results suggest that OsWRKY114 promotes JA responses by inhibiting *JAZ* gene expression.

## 3. Discussion

Plants can now be engineered using a variety of advanced biotechnological techniques to develop new crop cultivars with stress resilience [33,34,35]. Therefore, identifying novel genes conferring BSR and understanding their molecular mechanisms are critical for plant breeding. Rice is one of the most consumed crops globally, and this consumption is expected to increase in the future due to the growing population [36]. In light of increasingly compromised rice yields due to rapid climate change, it is urgent to enhance rice production by developing cultivars that resist destructive pathogens such as *Xoo* and *F. fujikuroi*.

We previously revealed that the rice transcription factor OsWRKY114 represses ABA signaling [24,25]. ABA is a major phytohormone that inhibits germination. Surprisingly, in the current study, *OsWRKY114_OX_* kernels germinated more slowly than wild-type kernels (Figure 1A). GA and ABA regulate germination antagonistically, and GA is required for germination [37]. Some OsWRKY transcription factors are known to simultaneously inhibit GA and ABA signaling. For example, overexpressing *OsWRKY24*, *OsWRKY53*, and *OsWRKY70* reduced both GA and ABA signaling [38]. Therefore, we examined whether OsWRKY114 is also involved in GA signaling. The transcript levels of *OsWRKY114* in wild-type plants were significantly reduced by GA treatment (Figure 1B), and GA-related genes (i.e., *OsGID1*, *OsXTH8*, *GA20_OX_2*, and *GA3_OX_1*) involved in GA signaling or GA biosynthesis were downregulated in *OsWRKY114_OX_* compared with wild-type plants (Figure 1C,D). These results suggest that overexpressing *OsWRKY114* represses GA signaling.

OsWRKY114 increases innate immunity against *Xoo* [23,24], and GA is important for susceptibility to *F. fujikuroi* [14,15]. Therefore, we examined whether OsWRKY114-mediated inhibition of GA signaling could improve innate immunity against *F. fujikuroi* in rice. Indeed, *OsWRKY114_OX_* plants were significantly more resistant to *F. fujikuroi* compared with wild-type plants (Figure 2A–C). The expression of *OsWRKY114* was significantly decreased by *F. fujikuroi* as well as GA (Figure 1B and Figure 2D). This result suggests that OsWRKY114 is negatively regulated by GA during *F. fujikuroi* infection. Moreover, JA-dependent defense responses are a major factor in immune responses against *F. fujikuroi* [14,15]. Thus, we measured the expression levels of JA-related genes in *OsWRKY114_OX_* and wild-type plants and found that *OsJAZ5*, *OsJAZ9*, and *OsJAZ14* transcripts were less abundant in *OsWRKY114_OX_* than in the WT (Figure 3B). A recent study showed that downregulating *OsJAZ* genes is important for resistance to *F. fujikuroi* [16]. Here, we showed that OsWRKY114-mediated repression of *OsJAZ* genes is important for innate immunity against *F. fujikuroi*.

In our current and previous study, we demonstrated that OsWRKY114 enhances resistance to *F. fujikuroi* and *Xoo* by modulating phytohormone signaling, such as GA, JA, and ABA signaling (Figure 3C). Since climate change is exacerbating plant damage caused by biotic stress, the development of crop cultivars with enhanced resistance to biotic stress is needed to help ensure global food security. This necessitates not only the identification of genes conferring BSR but also an understanding of their regulatory mechanisms. Therefore, further experiments should be conducted to examine resistance to other pathogens as well as transcriptomic changes via deep sequencing. The current study provides valuable information for further research on the detailed mechanism of the role of OsWRKY114 in BSR.

## 4. Materials and Methods

### 4.1. Plant Materials and Growth Conditions

Rice (*Oryza sativa* L. cv. Ilim) kernels were used as the wild type in this study. The *OsWRKY114_OX_* lines were generated and confirmed previously [23]. All kernels were sterilized with a 2% sodium hypochlorite solution and rinsed with sterile distilled water. The kernels were germinated in sterile distilled water for 5 days, transferred to Murashige and Skoog (MS) medium, and grown under a 16 h light/8 h dark photoperiod at 28 °C.

### 4.2. Germination Analysis

Germination rate was assessed using 36 kernels each from *OsWRKY114_OX_* and wild-type plants. Kernels were germinated for 6 days on half-strength MS medium in a growth room under continuous light at 28 °C with 40% relative humidity.

### 4.3. Gene Expression Analysis

Plants were frozen and ground to powder in liquid nitrogen. Total RNA was extracted from the leaves using an RNeasy Plant Mini Kit (QIAGEN, Germantown, MD, USA) according to the manufacturer’s instructions. cDNA was synthesized using SuperScript III reverse transcriptase (Invitrogen, Waltham, MA, USA). RT-qPCR was performed (with primers listed in Table S1) as previously described [25]. Gene expression was quantified using the comparative Ct method. *OsActin* was used as an internal control to quantify relative gene expression [39].

### 4.4. Bakanae Disease Assay

The bakanae disease assay was performed as previously described [40]. Briefly, *F. fujikuroi* CF283 was cultured on potato dextrose agar medium (200 g/L potato infusion, 20 g/L dextrose, and 20 g/L agar), and the fungal spores were collected and adjusted to a final concentration of 1 × 10^5^ spores/mL. *OsWRKY114_OX_* and wild-type plants were grown on sterilized filter paper moistened with distilled water. After 4 days, the seedlings were immersed in fungal spore suspension at 28 °C for 24 h and grown in MS medium without sucrose. The disease index and survival rates of wild-type and transgenic plants were analyzed at 21 DAI. The disease index scale ranged from 0 to 3 as follows: 0, no symptoms appeared; 1, blight symptoms appeared on leaf tips; 2, shoots wilted and leaf color changed to pale green; and 3, the whole leaf dried out.

### 4.5. Statistical Analysis

All experiments were independently conducted at least three times, and the average values from the independent experiments are presented. The data were analyzed by *t*-test. Asterisks denote significant differences (* *p* < 0.05, ** *p* < 0.01).

## Data Availability

The data presented in this study are available in the article or the Supplementary Materials.

## Supplementary Materials

The following are available online at https://www.mdpi.com/article/10.3390/ijms24076604/s1.
