# Supplementary material for: OsWRKY114 Is a Player in Rice Immunity against Fusarium fujikuroi
Source: Int J Mol Sci. 2023 Apr 1;24(7):6604. doi: 10.3390/ijms24076604 (PMC10094899; doi:10.3390/ijms24076604)
Supplement: Supplementary file 1 [file ijms-24-06604-s001.zip › ijms-2302109-supplementary.pdf]

Gene	Locus number	Primer sequence	Purpose
OsWRKY114	Os12g02400	F: CACCCATGTTCCAAGTGACA	qRT-PCR
		R: ATCGTCAGGGTGACCATTTG	qRT-PCR
OsGID1	Os05g33730	F: CGGACGGGACGTTCGAG	qRT-PCR
		R: TATGATCACCGGGAACGGCT	qRT-PCR
OsXTH8	Os08g13920	F: TACAGCACCAACGCGTGCGT	qRT-PCR
		R: GTAGGACATGTAGTTGCGCTCGGCC	qRT-PCR
OsGA20$_{OX}$*2*	Os01g66100	F: GACTACTTCTTCAGCACC	qRT-PCR
		R: CTTCATCTCCTCGCAGTA	qRT-PCR
OsGA3$_{OX}$*1*	Os05g08540	F: GCTGTAAGGGATAAGTTGTT	qRT-PCR
		R: ACTCTCCTTGTCCTCTTC	qRT-PCR
OsMYC2	Os10g42430	F: TGGAGATCGAGGCCAAGATC	qRT-PCR
		R: TCTACTCGCAGGACCAGCTCAA	qRT-PCR
OsAOC	Os03g32314	F: CTCAACCAGATCGTCTTCCC	qRT-PCR
		R: GAAGTTGTTGAGGCAGGCGT	qRT-PCR
OsJAZ5	Os04g55130	F: TGTGTGCTTCACAGTATTTCG	qRT-PCR
		R: GGACAGAAGCAGCTCCAATG	qRT-PCR
OsJAZ9	Os03g08310	F: CGTCTGCGATTTGAGAATTG	qRT-PCR
		R: ATGCGACGAGAACCATCTTC	qRT-PCR
OsJAZ14	Os10g25250	F: CGATTCAAGGCCAATTTACTG	qRT-PCR
		R: AACAAGACGATTTCGCCAAC	qRT-PCR
OsActin	Os03g50885	F: CATTGGTGCTGAGCGTTTCC	qRT-PCR
		R: CTCCTTGCTCATCCTGTCAGC	qRT-PCR

Supplementary Table S1. Sequence of primers used in this study.
